# Mutant Parkin Impairs Mitochondrial Function and Morphology in Human Fibroblasts

**DOI:** 10.1371/journal.pone.0012962

**Published:** 2010-09-27

**Authors:** Anne Grünewald, Lisa Voges, Aleksandar Rakovic, Meike Kasten, Himesha Vandebona, Claudia Hemmelmann, Katja Lohmann, Slobodanka Orolicki, Alfredo Ramirez, Anthony H. V. Schapira, Peter P. Pramstaller, Carolyn M. Sue, Christine Klein

**Affiliations:** 1 Section of Clinical and Molecular Neurogenetics, Department of Neurology, University of Lübeck, Lübeck, Germany; 2 Department of Neurogenetics, Kolling Institute of Medical Research, Royal North Shore Hospital and University of Sydney, Sydney, New South Wales, Australia; 3 Institute for Medical Biometry and Statistics, University of Lübeck, Lübeck, Germany; 4 Department of Clinical Neurosciences, Institute of Neurology, University College London, London, United Kingdom; 5 Institute of Genetic Medicine, European Academy, Bolzano, Italy; National Institutes of Health, United States of America

## Abstract

**Background:**

Mutations in *Parkin* are the most common cause of autosomal recessive Parkinson disease (PD). The mitochondrially localized E3 ubiquitin-protein ligase Parkin has been reported to be involved in respiratory chain function and mitochondrial dynamics. More recent publications also described a link between Parkin and mitophagy.

**Methodology/Principal Findings:**

In this study, we investigated the impact of *Parkin* mutations on mitochondrial function and morphology in a human cellular model. Fibroblasts were obtained from three members of an Italian PD family with two mutations in *Parkin* (homozygous c.1072delT, homozygous delEx7, compound-heterozygous c.1072delT/delEx7), as well as from two relatives without mutations. Furthermore, three unrelated compound-heterozygous patients (delEx3-4/duplEx7-12, delEx4/c.924C>T and delEx1/c.924C>T) and three unrelated age-matched controls were included. Fibroblasts were cultured under basal or paraquat-induced oxidative stress conditions. ATP synthesis rates and cellular levels were detected luminometrically. Activities of complexes I-IV and citrate synthase were measured spectrophotometrically in mitochondrial preparations or cell lysates. The mitochondrial membrane potential was measured with 5,5′,6,6′-tetrachloro-1,1′,3,3′-tetraethylbenzimidazolylcarbocyanine iodide. Oxidative stress levels were investigated with the OxyBlot technique. The mitochondrial network was investigated immunocytochemically and the degree of branching was determined with image processing methods. We observed a decrease in the production and overall concentration of ATP coinciding with increased mitochondrial mass in *Parkin-*mutant fibroblasts. After an oxidative insult, the membrane potential decreased in patient cells but not in controls. We further determined higher levels of oxidized proteins in the mutants both under basal and stress conditions. The degree of mitochondrial network branching was comparable in mutants and controls under basal conditions and decreased to a similar extent under paraquat-induced stress.

**Conclusions:**

Our results indicate that *Parkin* mutations cause abnormal mitochondrial function and morphology in non-neuronal human cells.

## Introduction

Mutations in the *Parkin* gene (MIM 602544) are the most common known cause of early-onset Parkinson disease (PD; MIM 168600), accounting for up to 77% of the cases with an age of onset <30 years [1]. *Parkin* encodes a 465-amino-acid protein with a modular structure [2], [3].

In addition to Parkin's roles as E3 ligase and neuroprotectant, it has been reported to be involved in mitochondrial function [4]. This connection was first established when *Parkin* loss-of-function mice presented with reduced expression of mitochondrial function- and oxidative stress-related proteins, decreased mitochondrial respiratory capacity and increased oxidative damage [5]. Similar results were obtained in additional animal models [6], [7], [8], [9].

Investigation of mitochondrial function in human samples supports the findings from animal studies. Functional assays in leukocytes as well as fibroblasts of patients with homozygous or compound-heterozygous *Parkin* mutations consistently showed reduced mitochondrial complex I activity, coinciding with reduced ATP synthesis rates [10], [11].

Another intriguing finding is that Parkin is involved in the regulation of mitochondrial morphology. The knockdown of *Parkin* causes swollen mitochondria in *Drosophila* indirect flight muscles [12], [13]. As a human model, fibroblasts from PD patients with *Parkin* mutation have been used to investigate mitochondrial morphology and revealed a greater degree of mitochondrial branching in the patients than in controls [11].

Recently, several publications linked Parkin and mitophagy in different cellular models [14], [15], [16], [17]. Induced by loss of mitochondrial membrane potential, Parkin is recruited by the PTEN-induced putative kinase 1 (PINK1; MIM 608309) to dysfunctional mitochondria, where it mediates their engulfment by autophagosomes and their selective elimination [14], [17], [18], [19]. In *Drosophila*, terminally damaged mitochondria are labeled for degradation by ubiquitylation of mitofusion (mfn) [20], [21], [22].

Most of the above-mentioned data explaining Parkin's function with respect to PD were either obtained in animal models or in small sets of human cellular samples. Here, we evaluated a larger sample of *Parkin*-mutant fibroblasts for changes in mitochondrial function and morphology.

## Materials and Methods

### Ethics statement

The study was approved by the ethics committee of the University of Lübeck and all participants gave written, informed consent.

### Patients

Skin biopsies were obtained from 11 individuals including six affected cases (mean age ± STD: 56.2±13.3 years) with two mutant *Parkin* alleles and five age-matched controls (mean age±STD: 51.8±11.5 years) without mutations in known PD genes. Phenotypic and genotypic data are summarized in Table 1 (further clinical details were published earlier [23]).

**Table 1:** Table 1. Genotypic and phenotypic characterisation of investigated individuals.

	Code	Sex	Age of onset (yr)	Age (yr)	Mutation	Zygosity	Clinical status
**Mutants**	B11	M	64	79	delEx7+c.1072delT	compound heterozygous	affected
	B125	M	43	62	c.1072delT	homozygous	affected
	B300	F	34	49	delEx7	homozygous	affected
	L3035	M	31	49	delEx3-4+duplEx7-12	compound heterozygous	affected
	L3048	M	15	57	delEx4+c.924C>T	compound heterozygous	affected
	L3244	F	37	41	delEx1+c.924C>T	compound heterozygous	affected
*∅* ± *STD*			*37.3*±*16.1*	*56.2*±*13.3*			
**Controls**	802.1	F	n/a	60	none	n/a	unaffected
	902.1	F	n/a	68	none	n/a	unaffected
	B963	M	n/a	44	none	n/a	unaffected
	B964	M	n/a	44	none	n/a	unaffected
	L3293	M	n/a	43	none	n/a	unaffected
*∅* ± *STD*				*51.8*±*11.5*			

### Tissue culture

Fibroblasts were cultured in high glucose Dulbecco's Modified Eagle's Medium supplemented with 10% foetal bovine serum and 1% penicillin–streptomycin (all PAA, Pasching, Austria) at 37°C, 5% CO_2_. In all assays, fibroblast passage numbers were matched (<15).

To induce oxidative stress, cells were treated with 2 mM paraquat for 24 h (Sigma-Aldrich, St. Louis, MO).

### Assessment of mitochondrial function

Cellular ATP synthesis rates were determined according to a published protocol [24]. In brief, the amount of protein was determined using the D*c* Protein Assay Kit (Bio-Rad, Hercules, CA, USA) following the manufacturer's instructions. Fibroblasts were harvested and diluted with cell suspension buffer (150 mmol/l KCl, 25 mmol/l Tris-HCl; pH 7.6, 2 mmol/l EDTA pH 7.4, 10 mmol/l KPO_4_ pH 7.4, 0.1 mmol/l MgCl_2_ and 0.1% [w/v] BSA) to a concentration of 1 mg protein per ml. ATP synthesis was initiated by the addition of 250 µl of the cell suspension to 750 µl of substrate buffer (10 mmol/l malate, 10 mmol/l pyruvate, 1 mmol/l ADP, 40 µg/ml digitonin and 0.15 mmol/l adenosine pentaphosphate). Cells were incubated at 37°C for 10 min. At 0 and 10 min, 50 µl aliquots of the reaction mixture were withdrawn, quenched in 450 µl of boiling 100 mmol/l Tris-HCl, 4 mmol/l EDTA pH 7.75 for 2 min and further diluted 1/10 in the quenching buffer. The quantity of ATP was measured in a luminometer (Berthold, Detection Systems, Pforzheim, Germany) with the ATP Bioluminescence Assay Kit (Roche Diagnostics, Basel, Switzerland) following the manufacturer's instructions. Cellular ATP levels were quantified in intact cells as described [25]. In both assays, the control average value per run was set to 100% and the relative average patient value was calculated. By this, variation of absolute ATP levels between experimental runs due to variable quality of the used kit was not taken into account.

To investigate respiratory chain function, mitochondria were isolated as published [25]. Mitochondrial respiratory chain complex activities were measured spectrophotometrically and expressed as ratios of citrate synthase activity [25].

The mitochondrial membrane potential was analyzed using 5,5′,6,6′-tetrachloro-1,1′,3,3′-tetraethylbenzimidazolylcarbocyanine iodide (Invitrogen, Carlsbad, CA) [25].

All measurements were performed in duplicate and in at least three independent runs per sample on a microplate reader (Synergy HT, BioTek, Winooski, VT).

### Quantification of oxidized proteins

Protein carbonyl levels were measured with the OxyBlot kit (Millipore, Billerica, MA), according to the manufacturer's recommendations. Carbonyl groups in the protein side chains were derivatized to 2, 4-dinitrophenylhydrazine (DNP). Western blot analysis was performed with an antibody against DNP. Equal loading was assessed using an antibody against mouse polyclonal anti-β-actin (Sigma-Aldrich, St. Louis, MO). The experiment was performed three times and a representative blot was analyzed densitometrically with TotalLab software (Nonlinear Dynamics, Newcastle, UK).

### Assessment of mitochondrial branching

The mitochondrial network in fibroblasts was stained with an anti-GRP75 antibody (Abcam, Cambridge, MA) in combination with the zenon immunolabelling kit (Invitrogen, Carlsbad, CA) according to manufacturer's protocol.

The mitochondrial network morphology was investigated using a fluorescence microscope equipped with an ApoTome and AxioVision software (all Zeiss, Jena, Germany). By means of ImageJ 1.42, raw images were binarized, mitochondrion area and outline were measured and the form factor was calculated (defined as [P_m_^2^]/[4πA_m_]), where P_m_ is the length of the mitochondrial outline and A_m_ is the area of the mitochondrion [11]). The form factor allows quantifying the degree of branching of the mitochondrial network. Images of at least five randomly selected cells per individual were analyzed under basal conditions and after paraquat treatment.

### Statistical analysis

The Mann-Whitney U test was applied for comparisons between mutants and controls. In case of the ATP concentration and synthesis data, a Mann-Whitney U test was performed to compare the mutant with the average control values set to 100% in each run. For evaluation of the impact of stress on cells, the Wilcoxon matched-pairs signed-ranks test was used to determine differences before and after treatment. The significance level was set at 0.05.

## Results

We analyzed primary dermal fibroblasts from six PD patients with homozygous or compound-heterozygous mutations and five age-matched controls for mitochondrial changes (Table 1).

### Respiratory chain function is impaired in *Parkin*-mutant fibroblasts

**Figure 1:**
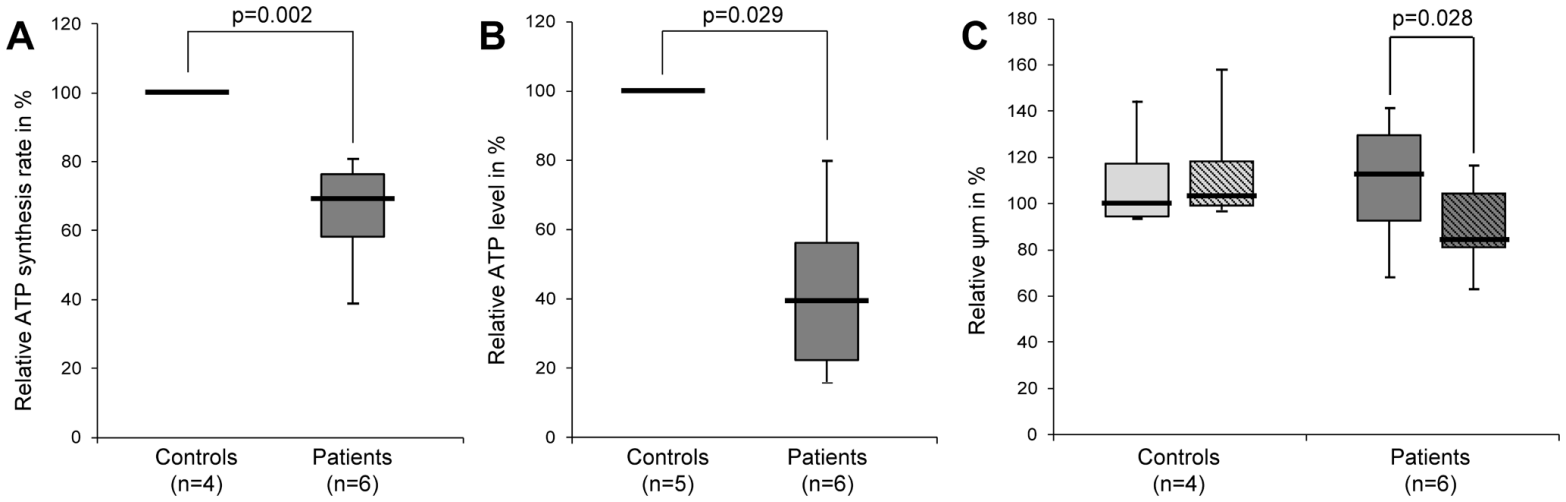
Mitochondrial function. (A) Basal ATP synthesis rates. The assay demonstrated a significant reduction in ATP production in the *Parkin*-mutant patients (median [IQR]: 39% [23%, 55%]) compared to controls (set to 100%). (B) Basal ATP levels. Quantifying the overall cellular ATP concentration showed significantly lower levels in the mutants (median [IQR]: 69% [58%, 75%]) than in controls (set to 100%). (C) Mitochondrial membrane potential under basal and oxidative stress conditions. Control (median [IQR]: 100% [94%, 115%]) and patient fibroblasts (median [IQR]: 113% [93%, 128%]) with *Parkin* mutations show similar membrane potential under basal conditions. When the cells were treated with paraquat (shaded boxes), no relevant changes were detected in the controls (median [IQR]: 102% [100%, 118%]). In the *Parkin* mutants, a significant drop of the membrane potential was observed (median [IQR]: 84% [81%, 103%]). The median, the interquartile range (IQR), the minimum and the maximum value of 6 (A) or 4 (B) independent experimental runs are plotted. In each experimental run the average ATP level in the controls was set to 100%.

We first determined ATP synthesis rates and cellular ATP concentrations. These experiments revealed a significant (ATP synthesis: p = 0.002; ATP level: p = 0.029) reduction of both parameters in mutants compared to controls (Figure 1A, B).

We next investigated whether the lower ATP synthesis rates and cellular ATP levels in the patient samples were due to a dysfunction of respiratory chain enzymes. Kinetic assays performed in mitochondrial preparations showed no significant differences in complex I activity in patient fibroblasts (median [interquartile range; IQR]: 72% [66%, 87%], n = 6) compared to controls (median [IQR]: 100% [80%, 102%], n = 5). Furthermore, we performed an NADH ferricyanide reductase assay, which allows to determine the content of functional complex I [26]. This assay also showed similar levels in mutants (median [IQR]: 87% [71%, 101%], n = 6) and controls (median [IQR]: 100% [88%, 107%], n = 5). The activities of complexes II+III (median [IQR]: patients, 134% [85%, 180%], n = 6; controls, 100% [92%, 112%], n = 5) and IV (median [IQR]: patients, 95% [82%, 108%], n = 6; controls: 100% [75%, 112%], n = 5) were comparable in *Parkin*-mutant fibroblasts and controls.

We then went on to determine the mitochondrial membrane potential as a central parameter of mitochondrial integrity. Under basal conditions, the membrane potential was similar in patients and controls, whereas under paraquat-induced stress, the mutants showed a significant (p = 0.028) loss (Figure 1C).

### Mutant-*Parkin* alters the cellular oxidative stress level

**Figure 2:**
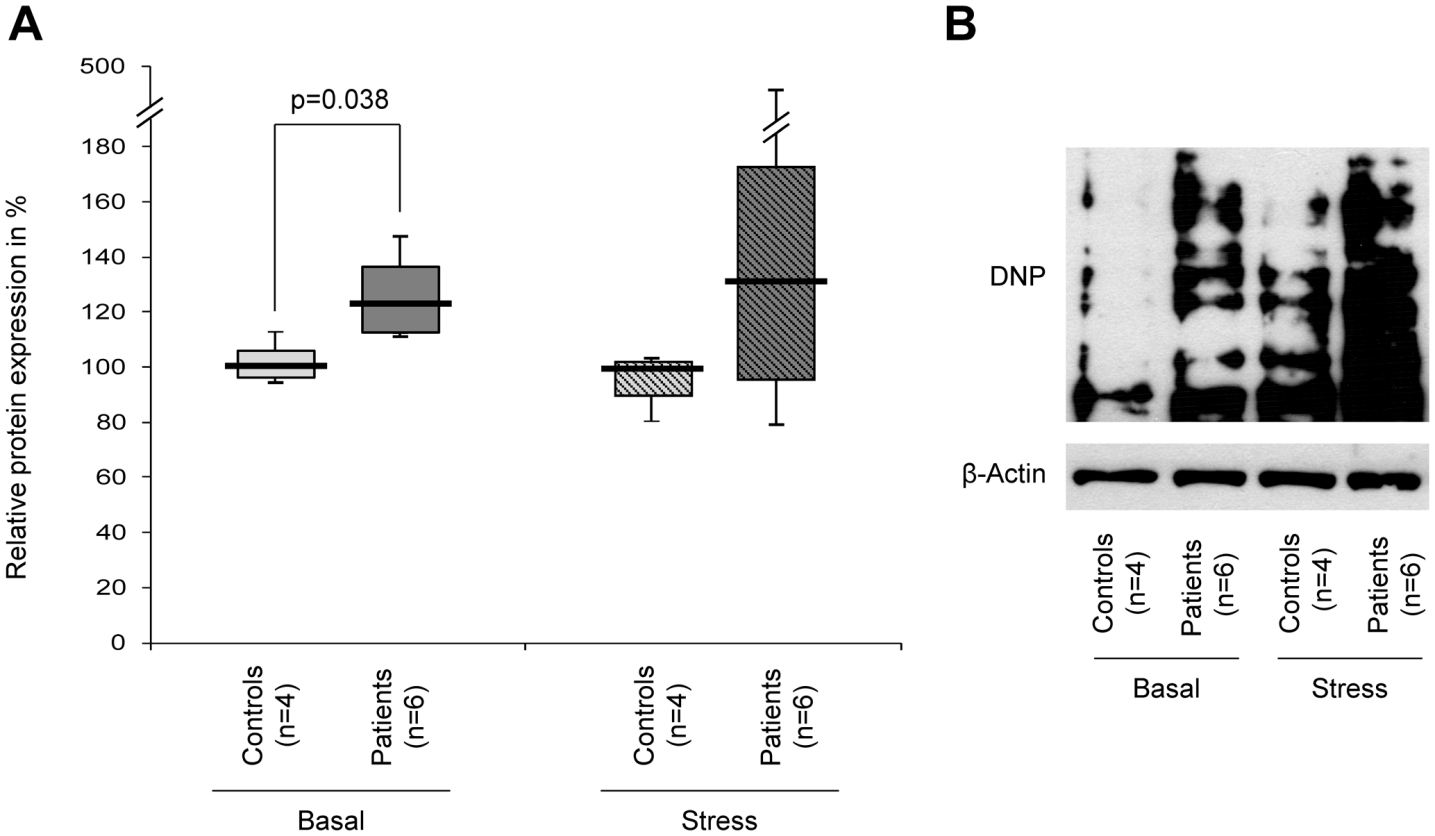
Protein oxidation under basal and stress conditions. Oxidation of proteins in *Parkin*-mutant fibroblasts and controls was determined by means of an OxyBlot. (A) When quantifying the protein oxidation in each individual using an antibody against DNP, the *Parkin* mutants (median [IQR]: 123% [113%, 136%]) showed significantly higher levels of oxidation than the controls (median [IQR]: 100% [97%, 105%]). After treatment of the cells with paraquat (shaded boxes), the difference in oxidation between mutants (median [IQR]: 131% [96%, 172%]) and controls (median [IQR]: 100% [90%, 102%]) increased, but was no longer significant. Equal protein loading was verified with an antibody against β-actin. Expression ratios of oxidized proteins vs. β-actin were calculated. The median, the interquartile range (IQR), the minimum and the maximum value of the investigated groups of individuals are given. (B) OxyBlot of pooled protein samples before and after paraquat treatment showing a similar trend as identified by individual measurements.

In order to determine basal levels of oxidative stress in fibroblasts from *Parkin* mutants, we applied an OxyBlot. This technique demonstrated significantly (p = 0.038) higher levels of oxidized proteins in the *Parkin*-mutant samples than in controls (Figure 2A). Under paraquat-induced stress, the difference in oxidation between mutants and controls increased markedly. Due to increased variability of the individual results after stress this result was not significant (Figure 2A). The findings from densitometric analyses in single individuals were supported by an OxyBlot performed with pooled control and patient samples under basal and stress conditions (Figure 2B).

### Mitochondrial mass is increased in *Parkin*-mutant fibroblasts

Next, we tested mitochondria of our fibroblast samples for morphological changes since impaired mitochondrial fission [11], [27], [28] or the failure to activate mitophagy [18] are well-established findings in *Parkin* null mutants.

**Figure 3:**
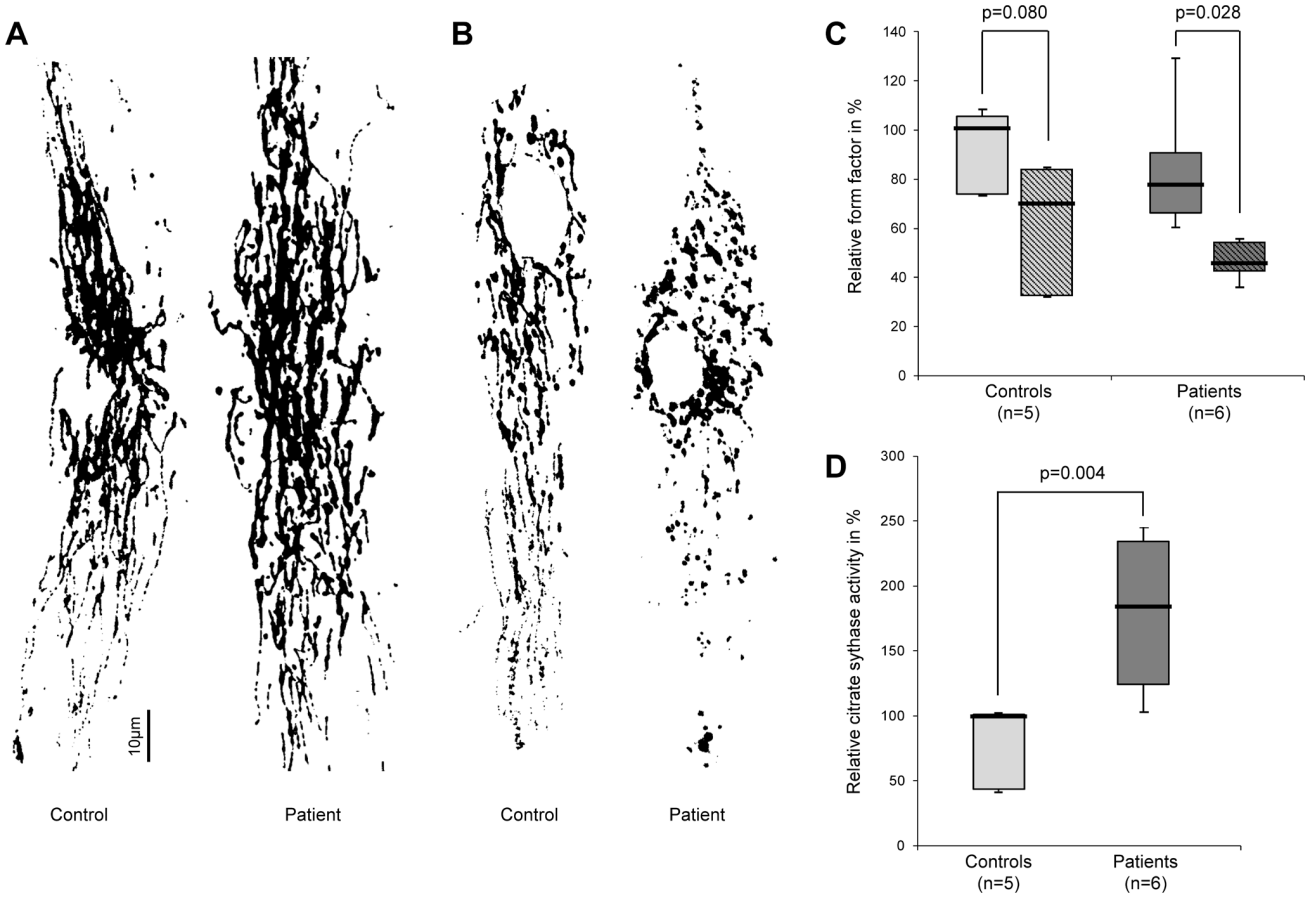
Morphology of the mitochondrial network. (A) Images of the mitochondrial network in control and patient fibroblasts demonstrating similar degrees of branching under basal culturing conditions. (B) After treatment with paraquat, the network was less branched in patients and controls. (C) The degree of mitochondrial branching (form factor) was comparable in patients (median [IQR]: 78% [66%, 90%]) and controls (median [IQR]: 100% [73%, 105%]) under standard cell culturing conditions. When treated with paraquat (shaded boxes), the form factor decreased significantly in the mutant samples (median [IQR]: 46% [43%, 54%]). By contrast, a drop seen in controls (median [IQR]: 70% [32%, 84%]) was not significant. (D) Citrate synthase activity in cell lysates. *Parkin* mutants (median [IQR]: 183% [125%, 232%]) showed significantly higher citrate synthase activities than controls (median [IQR]: 100% [43%, 101%]), indicative of increased mitochondrial mass per cell in the former. Citrate synthase activity in cell lysates was normalized for protein concentration. The median, the interquartile range (IQR), the minimum and the maximum value of the investigated groups of individuals are shown.

To compare the degree of mitochondrial network branching in *Parkin* mutants and controls, we determined the form factor. This morphological assessment demonstrated no differences between mutant and control individuals under basal conditions (Figure 3A–C). After treatment with paraquat, the degree of branching decreased by 34% within the controls and by 46% within the *Parkin*-mutant samples. This drop was only significant (p = 0.028) in the latter group (Figure 3A–C).

Finally, to quantify the mitochondrial mass per cell, we determined the citrate synthase activity in cell lysates. These levels were significantly (p = 0.004) higher in mutants than in controls (Figure 3D).

## Discussion

In this study we demonstrate that mutations in *Parkin* cause abnormal mitochondrial function and morphology in PD patient fibroblasts.

Oxidative stress is a key element implicated in the pathophysiology of PD, as recently further supported by studies on human skin fibroblasts from PD patients [11], [25]. Our results demonstrate increased oxidative stress levels in *Parkin-*mutant fibroblasts under basal conditions. This difference between mutants and controls became more pronounced when the cells were exposed to paraquat. In keeping with our findings, a deficiency of the Parkin interaction partner PINK1 has been reported to cause mitochondrial accumulation of calcium in mammalian neurons, resulting in a mitochondrial calcium overload which then stimulates the production of reactive oxygen species (ROS) via NADPH oxidase [29].

Furthermore, there is strong evidence that a deficit in respiratory chain function is involved in the pathogenesis of PD [30], [31]. In a study on *Parkin*-mutant fibroblasts, the authors reported that a decrease in ATP production was linked to complex I [11]. Here, we determined significantly reduced ATP synthesis rates and cellular concentrations in patient cells with *Parkin* mutations compared to controls. By contrast, we saw no significant difference in complex I activity between patient samples and controls. Also the activities of complexes II to IV were not significantly altered in the *Parkin*-mutant cells. A possible explanation for this discrepancy might be a loss of the electron carrier glutathione or oxidation of ubiquinone due to increased oxidative stress in the patient cells.

When quantifying the mitochondrial membrane potential as a central factor of mitochondrial integrity [14], we found no impairment in the *Parkin* mutants under basal culturing conditions. However, exposure to high levels of ROS caused a significant decline of the membrane potential in these cells. In an earlier study, the membrane potential was found to be decreased in *Parkin*-mutant fibroblasts already under basal conditions and culturing in glucose depletion medium supplemented with galactose further worsened the situation [11]. If the mitochondrial membrane potential data were corrected for mitochondrial mass per cell, a similar outcome would be expected in our study.

Recently, Parkin has been shown to act downstream of PINK1 in a common pathway which appears to regulate mitochondrial morphology [27], [28], [32]. Two studies in human cells also demonstrated an impact of mutations in *Parkin* [11] and *PINK1* [25] on the shape of the mitochondrial network. *Parkin*-mutant cells were found to be more prone to enter fusion as reflected by a significant increase in mitochondrial branching in the patient group [11]. By contrast, we detected no significant differences in the degree of branching between *Parkin* mutants and controls under basal conditions but an increase in mitochondrial mass in the former. In the above-mentioned study [11], fibroblasts were exposed to rotenone, an inhibitor of the respiratory chain complex I. This treatment induced mitochondrial fragmentation in *Parkin*-mutant and control cells to a comparable extent. Similarly, in our study, no significant differences in branching between mutants and controls were detected after exposure to paraquat.

In light of the most recent publications ascribing Parkin a role in promotion of mitophagy [14], [15], [16], [17] our results can be interpreted as consequences of the inability of mutant Parkin to perform its function. In *Drosophila*, the initiation of mitophagy depends on ubiquitylation of the fusion factor mfn by parkin. Following mfn ubiquitylation, dysfunctional mitochondria are prevented from re-fusion with functional mitochondria [20], [21], [22]. If this ubiquitylation is impaired in *Parkin* mutants under stress, one could imagine that mitochondria with disturbed respiratory chain function are no longer separated and eliminated from the general pool but dominate cellular (dys)function. This effect is in keeping with decreased ATP synthesis rates, elevated oxidized protein levels, increased mitochondrial mass and the observed stress-induced loss of mitochondrial membrane potential in the patient fibroblasts investigated here. Furthermore, one would expect that due to impaired Mfn1/2 deactivation/degradation, mitochondria should be less fragmented in *Parkin*-mutant than in control cells under stress conditions. Since mitochondrial fusion and fission are transient events, dynamic techniques to quantify the degree of mitochondrial branching would be preferable to the method established so far [11]. Methodological restrictions together with great inter-individual variations in branching especially after exposure to mitochondrial stressors render it impossible to detect subtle morphological differences between mutants and controls.

In the future, further investigations of the relationship between changes in mitochondrial dynamics/turnover and cell death will be necessary to identify potential targets for neuroprotective drugs redirecting *Parkin*-mutant cells towards survival. The results from our study underline that also non-neuronal cells, such as fibroblasts from patients, allow important insights into the mechanisms underlying PD and, therefore, will be a useful tool to pursue this scientific goal.
